# Supplementary material for: “If it wasn’t forced upon me, I would have given it a second thought”: Understanding COVID-19 vaccine hesitancy in an outlier county in the Bay Area, California
Source: PLoS One. 2023 Dec 21;18(12):e0290469. doi: 10.1371/journal.pone.0290469 (PMC10735040; doi:10.1371/journal.pone.0290469)
Supplement: S1 File — (PDF) [file pone.0290469.s002.pdf]

“If it wasn’t forced upon me, I would have given it a second thought”: Understanding COVID-19 Vaccine Hesitancy in an Outlier County in the Bay Area, California

INITIAL CODEBOOK

Theme	Code	Example	
**Reasons for not getting vaccinated**			
**Individual Reasons (Beliefs, Attitudes, Knowledge)**			
	Personal beliefs - don’t need it (I can handle covid) and<br>People can fight off covid without the vaccine	“I'm not diabetic. I'm not obese. My blood pressure is fine, my heart is fine, so I didn't have comorbidities that would lead me to a place where I think I should really get this.” -132<br><br>“I would rather depend on my own immune system and feeding my body the right things in order to ward off disease you know in general, even if it's COVID or whatever in general. this is how I strongly feel, and I've always felt for years.” -111	
	Personal beliefs - treatment options available	“if I were to catch COVID, it would not be good because of my asthma. And that scares me. But I think in the back of my mind, because my doctors told me, I qualify for the antibody treatment. If I were to catch COVID, that makes me feel better.” - 108	
	Personal beliefs - alternative preventative practices	“I’ve been wearing my mask I’ve been protective myself, and doing what I’m supposed to do following the protocols about like only going out when I needed to. Um so then my daughter when she goes to daycare wear masks and you know make sure shes protected `so I I mean I feel like I still follow protocols so why do I need to put a needle in my arm.” -106<br><br>“If I feel like I’m coming down with the cold, I just do things that she taught me. I go get garlic, ginger, lemon. Different kinds of things. Herbs that we get from Jamaica or we get from Jamaican	

		grocery stores. It works. I don't like pharmaceutical drugs unless I have to. Basically it's a second resort, not the first resort." -102	
	Medical Reasons - Side Effects	"I'm concerned that people aren't being informed about what actually is in it, what the side effects are. I know for a fact that it causes blood clots. My mother had her booster and a week after having a booster, ended up having a stroke. And her doctor told her it was from her booster. And that she is not to take any more of it." -127	
	Medical Reasons - Concern for Kids/Pregnancy	"my son is four years old right now, but he'll be turning five in July. So it just makes me scared on if I get him the vaccine, you know, how, you know, how will his body take it? How? You know, there's not any it's not studies, I feel like aren't I aren't far out enough and don't have like the years of, of studies behind the vaccine that I just feel like scared on on the way that it'll, it might affect my son." -114	
	Medical Reasons - interactions with Previous Illnesses	"I don't know if the vaccine will help with those health problems or make them worse. Would it be good for me? I'm young and I have problems and I'm trying to get rid of these health problems. Nip them in the bud while I can. So I just don't think I need to get vaccinated." -106	
	Religious Beliefs	"That really, in my heart, I believe God was protecting me and protecting my family protecting my children. Just not to just do something and jump on board when they have you know, absolutely no information and to more trust in God, trust in my prayers." -105	
**Interpersonal Reasons (Relationships and Network)**			
	Stories of Side Effects / Bad Outcomes	"Because the other thing is my wife's cousin who is post-menopausal. She's 60 years old. She had a period for a month after mRNA injections. So, I	

		mean it's not like I just read about it. I actually know two people that that happened to." -132	
	Skeptical of Vaccine and Booster Effectiveness - Boosters	"Everybody was so desperate for like a remedy that everybody just kind of took that information like oh, it's gonna protect me from COVID and I'm not gonna get it anymore, which in reality that wasn't true. And then they backed backed up and said, Okay, now the well that's not true, actually. You can get it and you're just gonna have one booster. Well no, now it's going to be like three boosters. So it's like, they don't even know what's going on. How am I supposed to be like, okay, yeah, I trust this thing 100% just shove this in my body." -124	
	Skeptical of Vaccine and Booster Effectiveness - The Vaccine is ineffective	"But then I heard that you can take the COVID shot and still get COVID. I don't really see a big reason for taking it when you could get the COVID shot and still catch it." -125	
**Structural Reasons (Systems, Services Deliver, Media and Policies)**			
	Mistrust of Healthcare Profession	"I mean the medical profession completely 100% abdicated their responsibility to care for people. To help them with preventative while they waited for this magic show to…unfurl. You know, and…and there are people who fell ill, who were frightened, who were frightened and intimidated. Who couldn't get access even to medical care even if they wanted to" -103<br><br>"And then there's also the mistrust, in a lot of communities of color just don't trust the healthcare system. Just a lot of historical implications. And just the level of healthcare in Vallejo. I don't know about Fairfield, but Vallejo, our healthcare system is pretty lackluster in terms, so just probably not rushing to go there." -102	
	Personal Beliefs - Don't	"...but if I say no, then just leave it at	

	want to be forced	that. Because then you just get annoyed and frustrated, and it's like someone who's like, “Here, have this food you don't like, or whatever.” And kind of shove it down your throat, or say religion, or something like that. Just to respect the other person's choice.” -129<br><br>“Why are you forcing people to do it? … I feel like I’m under so much control all of the time that this was the my one chance to put my nose up… ” -121<br><br>“I don’t like to be forced into it or coerced. So, if they were able to make me think like it was my idea “Maybe I should go get the vaccine”, then maybe I would have been quicker to get it. But since it was like “You need to get the vaccine…it’s like teenagers, right? You tell them go left, they go right. If it wasn’t forced upon me, I would have given it a second thought.” -101	
	Bodily Autonomy	“It's my decision to do what I want to do with my body. And what I wanted to do with my body is my decision.” -129	
	Mistrust of Information from Media, Government and Science Community - insufficient research on the vaccine	“the vaccine had just come out within a year, they expedited the process and they pushed it through. I think at some point in time it was considered an experimental drug by the FDA. It was just the fact of we, we as a society, we don’t know the long-term lasting effects of it. And that scares the crap out of me.” 104	
	Mistrust of Information from Media, Government and Science Community - didn’t have enough info	“It was like, you need this to protect yourself. And it was like, okay, well, why you know, why was that, there was just, there was no information behind it. So I couldn't go off anything medically, but being told, it'll protect you, it'll be good for you. And I was like, well, how do we really know this? You know, there was just no clear medical information that anybody could really give me.” -105	

	Mistrust of Information from Media, Government and Science Community - Inconsistent Public Messaging	"It's never been uniform. My sister in Idaho. Nobody was wearing masks. No people doing anything I don't know be with ring nobody was doing anything. And then here we're in basic lockdown. So, what really going on? Why do we need to be in lockdown, and nobody else does?" -128<br><br>"I hear them go back and forth on what they say. And one press conference, they're saying this, and then a month later, they're totally going against what they said at the last one. And it's like, it's hard to believe, people that aren't consistent with research or news or what's happening or what's going on, or what we should or shouldn't be doing, or the fourth booster or the fifth. I mean, everything, it's just become such a free for all. And he said this, and she said, It's just hard to believe anybody these days." -108	
	Mistrust of Information from Media, Government and Science Community - perceived misinformation	"they need the true science behind it. Not from the CDC. More from Harvard, Yale, New Haven. The scientists who are actually studying it, who give the truth about what's going on about the vaccine. I believe too many people are white washing everything and not really giving the general public the truth about it." -127	
	Mistrust of Information from Media, Government and Science Community - corruption of government/ big pharma	"You can't just bend back and forth for financial reasons or political reasons. And I just feel like all these boosters and it's an opportunity for big pharma to keep making more money. And although COVID is real, it's still giving them an opportunity to make more money. The more doses you can push out, the more money you're gonna get." -102	
	Mistrust of Information from Media, Government and Science Community - persuasion tactics seem	"Yeah, another another place offered like 50 bucks to take the vaccine for you to get the shot. … just as way to make people take the shot. Just a way to sucker people in to take the shot." -125	

	like a scam	“And then I just felt like all the incentives that were going on around “Oh, here’s a quesadilla or here’s a marijuana joint”. It just made it seem like a joke to me. You have all these other health concerns and crises going around, whether it’s diabetes, high blood pressure, HIV, STD’s. And there’s no health incentive to encourage people in those areas, but for this vaccine, they’re doing it for some money, so that was kind of weird.” -102	
**Consequences of Remaining Unvaccinated**			
**Individual Consequences (Beliefs, Attitudes, Knowledge)**			
	Second Guessing/ Mental Battle	“A lot, especially pressure from like, you know, like the media pressure from people and wanting like people to be vaccinated, including, you know, like people telling me I should be vaccinated. And for me choosing not to and still feeling pressured by people to tell me that we need to be vaccinated. So, in that way, I'm impacted because I feel pressured and and just insecure about about what I should do, in regards to like vaccination.” -116<br><br>“I'm having to question my decision as a parent, because of these rules that are being placed on me. … It's a mental battle with am I doing the right thing? And am I doing what's in the best interest of me and my family.” -108	
	Disclosure issues	“I think it's something that people have kind of treaded lightly talking about. We don't advertise whether we are or not. Sometimes we'll have those conversations with a few people, and that it's more low key. I'm not out there being an anti-vaxxer, and saying, don't get vaccinated. I'm not. It's just that it’s a personal decision and I think it's up to everyone what they want to do.” -128<br><br>“She goes, "You're vaccinated right?	

		You got your shot. You guys are vaccinated?" … I lost sleep over this, because, you know, it is dishonest. But [I said], "Yeah, yeah, you know. yeah". And she was like, "Okay, good, good." And I thought, … would she have asked me to leave? If I said no." -108	
	Self-Perception - not feeling heard	"We almost need a voice now, you know as crazy as that sounds, like oh the vaccinated versus the vaccinated. But it really feels like that. It really feels like we're living in a time where the community is now, or the world even, is divided by vaccinated verse unvaccinated. It's all over social media. It's this thing and i'm just like man. We need a voice. We need people to speak up, and actually hear us out and hear our beliefs in our position on this." -111	
	Self-Perception - not an 'anti-vaxxer"	"I think it's something that people have kind of treaded lightly talking about. We don't advertise whether we are or not. Sometimes we'll have those conversations with a few people, and that it's more low key. I'm not out there being an anti-vaxxer, and saying, don't get vaccinated. I'm not. It's just that it's a personal decision and I think it's up to everyone what they want to do." -128	
	Feeling like a minority	"It seems I'm always hearing most people are vaccinated. I always feel like the odd ball out because I always hear that most people are vaccinated these days." -111	
**Interpersonal Consequences (Relationships and Network)**			
	Conflict with Family, Friends and Colleagues - Couldn't see family	"my grandparents actually just came over for the first time… in two years. So me and my kids have not seen them, and they're older. … it was them asking us not to come around. And I respected that, you know, they are vaccinated, they're older, and I could only respect their wishes." -105	
	Conflict with Family,	"I've had several heated discussions	

	Friends and Colleagues - Pressure	with family members about it. I just feel like your choice is your choice. You can't make me do something because you wanted to do it and you feel like it's right for you. I have to do what's right for me. Where I feel like, me and my aunt (inaudible). They argue on the phone, maybe an hour because she got the shot and I didn't get it. And she wanted to know why and I kept explaining to her, like I'm explaining to you why. But for some reason, she just could not understand it. And then she told me she thought that was selfish." -109	
	Conflict with Family, Friends and Colleagues -stigma	"I had family members that were always "Don't come around me" and "Are you vaccinated?". It was crazy. Even sometimes you go around certain people and they'll say "Are you vaccinated" and I'm like "No." And they're like "Go get a mask."" -123	
	Treated differently socially (Perceived as stupid, uncaring or selfish)	"just the constant barrage of making those of us that decided not to get the vaccine making us feel bad about our decisions. It was interesting to see how, especially in the media, how we were painted to be people that were either misinformed or just downright stupid" -122<br><br>" There are people that think I'm evil and I'm selfish. They may not know me personally, but anyone who chooses not to get vaccinated doesn't care about their fellow man. Like those things. I mean on social media, I see it." -107	
	Treated differently socially - Looked down on for being unvaccinated	"when they found out I was unvaccinated, didn't want to be around me. Thought it was crazy. They would just give me these looks or else not being allowed to go into businesses, because they thought I was. Being unvaccinated, is not equally to being sick and being a carrier, I mean I'm not Typhoid Mary. I wore mask, get my distance, wash my hands, sprayed everything down, did everything else but	

		get a vaccine." -132	
**Structural Consequences (Systems, Services Deliver, Media and Policies)**			
	Treated differently by healthcare providers -pressure	"I did go see an optometrist while I was unvaccinated. He kind of gave me that attitude like you're not vaccinated? I strongly urge you to get vaccinated. Kind of looking down at you. You didn't do what you were supposed to do." -102	
	Faced Discriminatory Policies (school field trips, workplace, restaurants and concert policies)	"I wasn't able to do anything because I wasn't vaccinated. I've been rejected at bars, clubs. Been rejected. Made complete plans and was rejected at the door because it didn't have enough information." -123<br><br>"I was trying to go see my wife at a nursing home. And they won't let me go see her unless I go get the vaccine." -131	
	Faced Discriminatory Policies - Work/income at risk	"There was a job a job I was interested in that it was like you got to take the shot to work here but I didn't agree with it so I didn't take the job." -125	
	Employment Implications (pressure and policies)	"because of the certain funding that we receive. It may say in that certain grant that in order for us to receive this grant that employees have to be vaccinated. So then that if if that would make me not eligible to work within their grant, that would make me lose my job." -16	
